# Human Scalp Hair as an Indicator of Exposure to the Environmental Toxin β-*N*-Methylamino-l-alanine

**DOI:** 10.3390/toxins10010014

**Published:** 2017-12-27

**Authors:** Simoné Downing, Laura Louise Scott, Nadezda Zguna, Timothy Grant Downing

**Affiliations:** 1 Department of Biochemistry and Microbiology, Nelson Mandela University, P.O. Box 77 000, Port Elizabeth 6031, South Africa; Simone.Downing@mandelda.ac.za (S.D.); Laura.Scott@mandela.ac.za (L.L.S.); 2 Unit for Analytical Chemistry, Department of Environmental Science and Analytical Chemistry, Stockholm University, SE 106 91 Stockholm, Sweden; Nadia.Kiselova@aces.su.se

**Keywords:** β-*N*-methylamino-l-alanine, BMAA, exposure, aerosol, diet, hair, bioaccumulation, cyanobacteria

## Abstract

Dietary or aerosol exposure to the environmental neurotoxin β-*N*-methylamino-l-alanine (BMAA) is a putative risk factor for the development of sporadic neurodegenerative disease. There are many potential sources of BMAA in the environment, but BMAA presence and quantities are highly variable. It has been suggested that BMAA in human hair may serve as an indicator of exposure. We sought to evaluate the use of the BMAA content of human scalp hair as an indicator of exposure, as well as the correlation between specific lifestyle or dietary habits, reported as hypothesised exposure risk factors, and BMAA in hair. Scalp hair samples and questionnaires were collected from participants in a small residential village surrounding a freshwater impoundment renowned for toxic cyanobacterial blooms. Data suggested a positive correlation between hair BMAA content and consumption of shellfish, and possibly pork. No statistically significant correlations were observed between hair BMAA content and residential proximity to the water or any other variable. Hair BMAA content was highly variable, and in terms of exposure, probably reflects primarily dietary exposure. However, the BMAA content of human hair may be affected to a great extent by several other factors, and as such, should be used with caution when evaluating human BMAA exposure, or correlating exposure to neurodegenerative disease incidence.

## 1. Introduction

The aetiology of sporadic neurodegenerative disease (sND) remains poorly understood, and with more than 95% of all neurodegenerative disease (ND) cases being of non-genetic or unknown cause, identifying risk factors has been the focus of many studies (reviewed in [1,2]). Unfortunately, to date only a handful of possible risk factors for amyotrophic lateral sclerosis (ALS) [1,2,3,4], Alzheimer’s disease (AD) [2,5] and Parkinson’s disease (PD) (reviewed in [2]) have been identified, with even fewer being formally recognised. A recurring trend is the link between place of birth and the development of sND [6], which supports an environmental aetiology, as well as the importance of exposure to a causative agent during early-life development [1,2,5]. However, to directly link exposure to an environmental toxicant to the development of ALS, AD or PD is extremely difficult, due to the long latencies of these diseases and the natural migration of people from their place of birth or early childhood years.

The non-proteinogenic amino acid β-*N*-methylamino-l-alanine (BMAA) has been proposed as a causative factor in the development of ALS, AD, and PD. BMAA is produced by cyanobacteria [7], and its presence has also recently been reported in diatoms [8,9] and dinoflagellates [10]. The environmental ubiquity of these producing organisms makes the risk of human exposure to this neurotoxin a global threat. While ongoing criticisms of the BMAA–neurodegenerative link have generated doubt of the actual role of BMAA in the aetiology of sND, new advances in the understanding of BMAA neurotoxicity have unequivocally validated the BMAA–neurodegenerative disease hypothesis. A recent report has emphasised that exposure to BMAA during specific critical and vulnerable developmental stages is a critical factor that will determine whether BMAA exposure will result in development of neurodegeneration [11]. 

As our understanding of BMAA toxicity expands, and the link between this environmental toxin and sND is strengthened, there is an increased necessity to gauge the risk of human exposure and to identify possible exposure risk factors, in order to avoid BMAA exposure during vulnerable or critical developmental periods. The need to identify and verify potential human BMAA exposure routes has persisted and, as a result, an extensive collection of data now exists that unequivocally confirms that humans, worldwide, are exposed to BMAA via dietary and aerosol exposure routes; specifically, these exposure routes include dietary intake of BMAA containing food items [12,13,14,15,16,17,18], and inhalation of aerosolised BMAA, either as cyanobacterial cells, water droplets [19,20,21,22], or cyanobacterial desert crust dust [23,24,25]. The amounts of BMAA reported in dietary items and aerosol show high temporal and spatial (geographical) variability; therefore, as is the case for most environmental toxicants, human exposure to BMAA is not uniform. Furthermore, additional factors potentially contribute to an individual’s susceptibility to BMAA toxicity and predisposition to development of sND, making direct relationships between BMAA exposure and development of sND difficult. 

Reports on hypothesised human BMAA exposure routes fall short, in that they describe only an indirect or anecdotal link between a specific source of BMAA and the presence of BMAA in humans following exposure, and none have confidently linked BMAA exposure to the development of sND in humans, a task that is extremely difficult, for reasons mentioned before. The occurrence of ALS “hotspots” across the world, in areas where cyanobacterial blooms persist, supports BMAA exposure as a risk factor in the development of this disease. Various reports have indirectly linked an increased risk of ALS development to potential BMAA exposure, either through the consumption of food items sourced from cyanobacterial bloom-containing waters, or via inhalation of or contact with cyanobacterial bloom material (reviewed in [4]). Caller et al. [26] observed a 25-fold increased risk of developing ALS in individuals that reside close to, and especially downwind of lakes (New Hampshire, United States of America) that frequently experience dense toxic cyanobacterial blooms.

Over the last three decades, reports on the presence and quantification of BMAA in humans have been limited to analysis of cerebrospinal fluid from ALS and control patients (*n* = 25) [27], the post-mortem analysis of brain tissues (*n* = 123) (reviewed in [28]), and one report on the presence of BMAA in human hair [29]. The latter report is the only study that attempted to directly link BMAA exposure (via the consumption of flying foxes known to contain BMAA [30]) to the presence of the toxin in humans. 

Considering the hypothesized long latency between exposure to BMAA and the development of sND, the ability to predict, monitor and prevent exposure is critical in managing disease incidence. The use of BMAA in human scalp hair as a potential marker that could indicate increased risk of sND development has been suggested [29]; however, the data on which this notion was based is anecdotal. The accumulation and metabolism of BMAA in humans remains poorly documented, with only a single report on the investigation of the presence BMAA in human hair [29]. Trace amounts of BMAA have been detected in Lesser Flamingo feathers following exposure to cyanobacteria [31], and a study in a non-human primate model showed a dose-dependent deposition of BMAA in hair following oral dosing [32]. The use of human hair to indicate and monitor BMAA exposure seems reasonable, but the suitability of this concept remains unverified.

Analysis of human scalp hair for the presence of environmental toxicants or narcotics (most common in this field) is commonly used, although this method has several advantages and disadvantages. Hair serves as an easily and unobtrusively obtainable biological matrix that can provide an indication of exposure over a very wide time window [33]. As toxicants enter the bloodstream, they are absorbed into the cells at the base of the hair follicle and become trapped inside the hair shaft during keratogenesis, where they remain with no further metabolism [33,34,35]. The presence of an environmental toxicant in human scalp hair can also be attributed to other non-systemic routes of acquisition, such as the deposition of toxicants onto the external surface of the hair via sweat and sebum, and via external contamination through air, water, or cosmetic treatments [33]. Factors like hair colour, cosmetic manipulations, and hair texture, which in turn is affected by chemical hair alterations and daily weathering of hair fibres (which increases with age), may all affect the associations of environmental toxicants with the hair fibre [35]. As human scalp hair growth is uniform, sectional hair analysis can provide useful information on environmental exposure [35]; however, when using human scalp hair as an indicator of exposure, identifying the specific route and time of exposure presents a challenge. 

The primary objectives of the current study were therefore to evaluate the use of human scalp hair BMAA content as a potential marker for BMAA exposure, and to attempt to correlate hair BMAA content with any of the proposed risk factors, such as residential proximity to persistent cyanobacterial blooms or consumption of any specific dietary item known to frequently contain BMAA. We emphasize that the study was not intended as an epidemiological study of sND, but was solely to determine whether there is a correlation between the BMAA content of human scalp hair and any specific lifestyle or dietary habit that has been reportedly hypothesized to lead to an increased risk of BMAA exposure or sND development. 

## 2. Results and Discussion

### 2.1. Surface Association of BMAA to Human Hair

All hair samples in this experiment were determined to be free of BMAA prior to exposure to BMAA. Stringent washing with 5% ethylene diamine tetraacetic acid (EDTA) and an organic solvent mixture (as described in Section 4.3.3) of BMAA-exposed hairs did not remove all surface-associated BMAA. Treated hair (frequently coloured with artificial dyes) retained approximately twice as much BMAA compared to virgin hair, after being washed with BMAA-containing water. These data are in accordance with various reports that have shown that chemical manipulation of hair, such as bleaching or dying, alters the crystalline structure of hair fibres, making them more accessible to external contaminants [35,36]. Other factors that may affect the structure of the hair fibre, like UV-radiation, heat, and weathering with age, can also have a considerable effect on the binding of exogenous BMAA to hair fibres. These data emphasize the fact that despite uniform exposure, retention of BMAA within scalp hair may vary considerably between individuals, due to differences in hair texture and condition.

### 2.2. BMAA in Human Scalp Hair

A total of 88 hair samples and corresponding questionnaires were collected from volunteer participants in Hartbeespoort, a small residential town surrounding the Hartbeespoort Dam (HBD) reservoir. Although every effort was made to ensure uniform gender distribution within the sample group, males (10 out of 88 hair samples) and females were not equally represented. The distribution of BMAA data is shown in Table 1.

Sixty-two percent of all samples tested positive for BMAA, with 25% of all positive samples containing only trace amounts of BMAA, below the limit of accurate quantification, and the remainder containing BMAA at quantifiable amounts, ranging from 0.006 to 9.6 µg BMAA g^−1^ hair. 

Although a total of 51 variables were considered in the questionnaire, emphasis here is given to specific variables previously reported as hypothesised potential risk factors for BMAA exposure, and consequently as potential factors related to an increased risk of the development of sND. The variables of particular interest were residential distance from the reservoir’s shoreline, the frequency in which the reservoir water was used in recreational activities, the frequency of consumption of fish sourced from the reservoir, and the frequency of consumption of shellfish.

Although this is the largest study of this nature undertaken to date, the sample size is still relatively small, and these data should serve only as an indication of possible correlations. In order to confirm these associations much larger sample sizes are required.

#### 2.2.1. Residential Proximity to the Water

**Figure 1:**
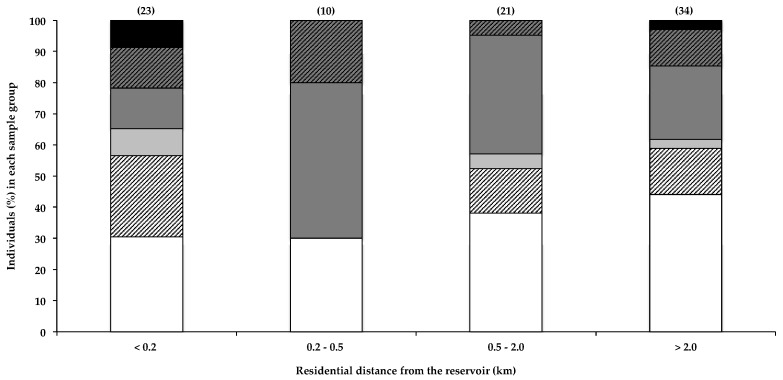
The presence of BMAA in individuals’ scalp hair and their residential proximity to the HBD reservoir. Data are depicted as the percentage of individuals in each distance category that falls within a specific BMAA quantity range: Not detected (white bars), Not quantified (cross-hatched white bars), >0.001 <0.01 µg BMAA g^−1^ hair (light grey bars), >0.01 <0.1 µg BMAA g^−1^ hair (dark grey bars), >0.1 <1.0 µg BMAA g^−1^ hair (cross-hatched dark grey bars), >1.0 µg BMAA g^−1^ hair (black bars). The sample size (*n*) of each category is given in parentheses.

Caller et al. [19] reported a marked increase in the risk of developing ALS in individuals that live within 800 m of a water body that frequently experiences toxic cyanobacterial blooms, which they postulate may result from BMAA exposure. In a recent survey, 77% of ALS patients were reported to have lived full-time, at some point during their lives, within 2 miles (3.2 km) of a water body, compared to 67% of survey participants that did not have ALS [4]. Furthermore, Banack et al. [22] reported the detection of trace amounts of BMAA in one of two air filters, used to filter air 3–4.5 m from the shoreline of a lake and pond with regular cyanobacterial blooms. Aerosolisation of BMAA from the HBD reservoir was recently investigated [21], and based on the amounts of BMAA detected in the aerosol, the authors concluded that exposure to BMAA via this route probably did not constitute a significant exposure risk. Data in Figure 1 show that the total percentage of BMAA-positive hair samples for individuals that live within 200 m of the water did not differ markedly from those that live more than 0.2 km away. Although Caller et al. [19] reported an increased risk of developing sND for residents living within approximately 800 m of water bodies, the potential for aerosol exposure decreases rapidly with distance from the shoreline [21], and the relatively low percentage of people living more than 20 km from the HBD, with BMAA in their hair, is therefore not attributable to differences in aerosol exposure. Figure 1 shows both the near-water (<200 m) group and the 200–500 m group for comparison. Most of the residential areas around the HBD reservoir lie downwind of the two prevailing wind directions (ESE and NW) [37], with only two participants reporting living in an upwind location. The data do not show any obvious correlations between residential proximity to the water and the amount of BMAA in the residents’ hair. Using the Fischer’s exact test, we compared the number of individuals that fall within each BMAA quantity range in the <0.2 km zone with that of each of the other distance categories, and we found no statistical significance (α < 0.05). As BMAA production by cyanobacteria is regulated by nitrogen availability [7,38], the presence of a cyanobacterial bloom does not necessarily always equate to the presence of BMAA in the water body or cyanobacterial scum, and therefore, BMAA exposure via aerosol would itself be highly variable.

#### 2.2.2. Frequency of Reservoir-Based Water Sports

The majority (>80%) of participants reported never using the reservoir for boating, swimming, fishing or water-skiing. The potential health risks associated with the cyanobacterial blooms on the HBD reservoir have been routinely publicized, and most residents are therefore aware of these risks. Only 9% of participants reported that they use the reservoir at least once a month for some water-related activity. The amounts of BMAA in the hairs of this 9% were not higher than those that did not use the reservoir for recreational activities. Although leading questions were avoided in the questionnaires, in this community the awareness of the health risks associated with cyanobacterial blooms has created a stigma that is attached to using reservoir water, and this may have discouraged participants from being completely forthcoming in their responses to questions pertaining to using the reservoir’s water. Bradley et al. [4] reported a three-fold association between the risk of developing ALS and water-skiing, and suggested that exposure to BMAA during water-skiing may account, as least in part, for this increased association. This could however not be substantiated by data from the current study.

#### 2.2.3. Consumption of Fish Sourced from the Reservoir

Relatively high consumption of fish harvested from the HBD reservoir by Hartbeespoort residents has been previously reported [38]. However only one of the participants of the current study reported the consumption of fish caught in the reservoir. Socioeconomic differences, and the stigma of using the HBD and its fish, could contribute to the fact that none of the participants reported eating fish from the reservoir.

#### 2.2.4. Consumption of Shellfish and Other Dietary Items

It must be emphasized that shellfish here refers to marine shellfish in general, and does not refer to that specifically harvested from the HBD reservoir. BMAA has been regularly detected in various species of shellfish frequently consumed by humans [12,13,14,15,16,17,18], and the farming and consumption of shellfish have been associated with an increased risk of developing ALS [16,39,40]. Consequently, the consumption of shellfish has been identified as a primary risk factor for BMAA exposure and potentially in the development of sND.

**Figure 2:**
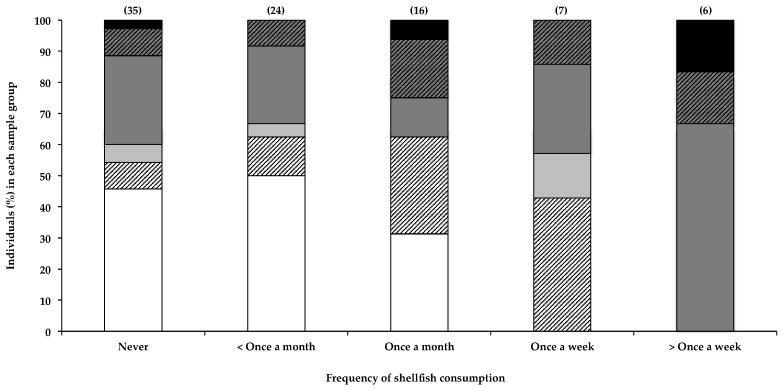
The presence of BMAA in individuals’ scalp hair, and the frequency with which they consume shellfish. Data are depicted as the percentage of individuals in each consumption frequency category that falls within a specific BMAA quantity range; Not detected (white bars), Not quantified (cross-hatched white bars), >0.001 <0.01 µg BMAA g^−1^ hair (light grey bars), >0.01 <0.1 µg BMAA g^−1^ hair (dark grey bars), >0.1 <1.0 µg BMAA g^−1^ hair (cross-hatched dark grey bars >1.0 µg BMAA g^−1^ hair (black bars). The sample size (*n*) of each category is given in parentheses.

Figure 2 shows the correlation between the consumption of shellfish and the presence of BMAA in human scalp hair. Data indicate a noticeable positive correlation between the frequency of shellfish consumption and the presence of BMAA in scalp hair. All individuals (*n* = 13) that reported eating shellfish once a week or more tested positive for BMAA in their scalp hair, whereas only half of individuals who ate shellfish less than once a month had BMAA in their hair. In order to determine whether the positive correlation between the frequency of shellfish consumption and hair BMAA content was statistically significant, we used the Fischer’s exact test to compare the number of individuals that fell within each BMAA quantity range in the group of individuals that ate shellfish less than once a month or never, with the number of individuals that fell within each BMAA quantity range in the group of individuals that consumed shellfish once a week or more, and in the group of individuals that consumed shellfish once a month or more. Statistical analysis confirmed that those that individuals who consumed shellfish less than once a month or never were significantly less likely to have BMAA in their hair, compared to those that consumed shellfish once a month or more (*p* = 0.0261) or once a week or more (*p* = 0.0055). These data therefore strongly support the consumption of shellfish as a significant BMAA exposure route. The observation that approximately 50% of individuals who reportedly never ate shellfish also tested positive for BMAA in their hair suggests additional sources of BMAA. The presence of BMAA in human hair in individuals that regularly consume shellfish is an interesting observation that supports literature on shellfish consumption as a credible BMAA exposure risk, and an observation that could benefit from further investigation, using much larger samples sizes.

**Figure 3:**
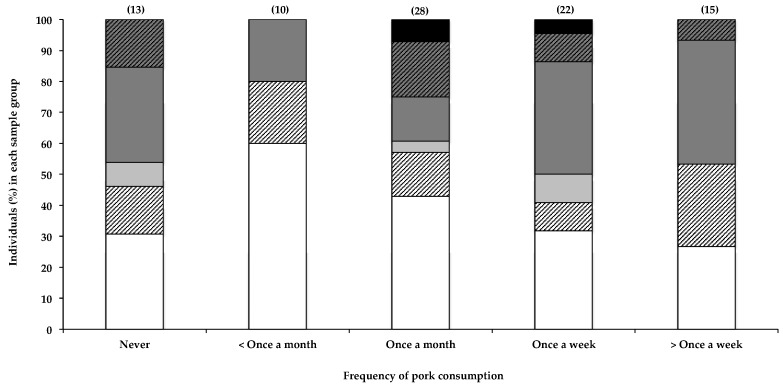
The presence of BMAA in individuals’ scalp hair and frequency with which they consume pork. Data are depicted as the percentage of individuals in each consumption frequency category that fall within a specific BMAA quantity range; Not detected (white bars), Not quantified (cross-hatched white bars), >0.001 <0.01 µg BMAA g^−1^ hair (light grey bars), >0.01 <0.1 µg BMAA g^−1^ hair (dark grey bars), >0.1 <1.0 µg BMAA g^−1^ hair (cross-hatched dark grey bars), >1.0 µg BMAA g^−1^ hair (black bars). The sample size (*n*) of each category is given in parentheses.

Interestingly, the only other seemingly positive correlation with the presence of BMAA in human scalp hair that was observed in this study was with the frequency in consuming pork (Figure 3), with 73% of individuals reportedly eating pork more than once a week testing positive for BMAA in their scalp hair, compared to 40% positive hair samples in those reportedly eating pork less than once a month. BMAA was also detected in hair of individuals (69%) who reportedly never consumed pork; however, as previously stated, this is to be expected, given the many potential sources of BMAA. Furthermore, pork is frequently included in processed meat products that may not be considered pork, *per se*, by some participants, resulting in the inaccurate or partial reporting of actual pork consumption by participants. The relatively small sample size did not allow for the separation of data based on consumption of each food item individually. The presence of BMAA in pork has not been previously reported, nor has the consumption of pork been identified as a possible risk factor for BMAA exposure. BMAA is known to accumulate through food webs, and there is a risk of bioaccumulation of this toxin in domestic livestock when BMAA-containing feed like fishmeal is used, or when cyanobacteria are present in drinking water.

Although statistical analysis of the data (as described in Section 2.2.4 and Section 4.4) did not show significance, data from this study may have potentially identified the consumption of pork as a previously unknown risk factor for BMAA exposure, an observation that would benefit from further investigation.

Furthermore, the accumulation of BMAA in crop plants irrigated with water containing BMAA or cyanobacteria has been reported [41]. The HBD supplies irrigation water to >150 km^2^ of agriculture. Residents of Hartbeespoort frequently consume locally-sourced fruits and vegetables, with 65% of participants reportedly eating locally-sourced crops once a week or more. However, no significant correlation between BMAA in scalp hair and the consumption of vegetables irrigated with HBD reservoir water was observed. 

### 2.3. Other Factors to Consider

BMAA in human scalp hair can accumulate via multiple routes, including both systemic deposition into the hair fibre, following an oral dose or inhalation of aerosol, or surface deposition, as a result of exposure of the hair surface to water or aerosol containing BMAA. Clearly this study cannot distinguish the source of the BMAA in the participants’ hair. Furthermore, the retention of exogenous and systemic contaminants within hair fibres is affected by the hair fibre structure and texture (condition of the hair), as well as individuals’ hair care habits and cosmetic manipulations. The latter two factors can either enhance or reduce the binding of contaminants to the hair fibre [35]. In addition, natural hair colour, which is determined by both the quality and quantity of melanins (eumelanin and phaeomelanin) in the hair shaft, is proposed as a factor that can significantly affect the accumulation of environmental toxicants within hair fibres [42]. 

**Figure 4:**
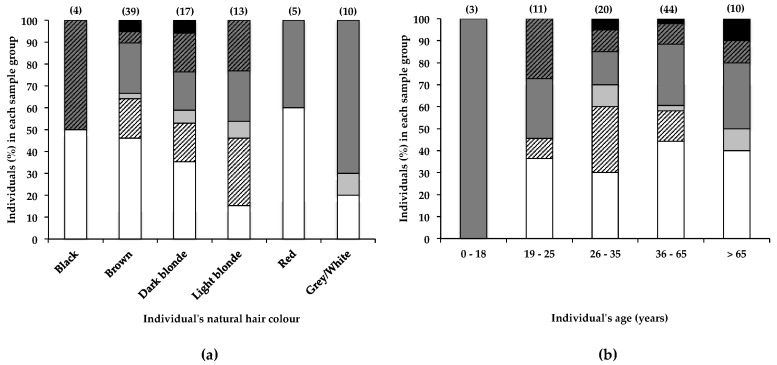
The presence of BMAA in individuals’ scalp hair and its correlation with the individual’s (**a**) natural hair colour and (**b**) age. Data are depicted as the percentage of individuals in each (**a**) hair colour category or (**b**) age category that falls within a specific BMAA quantity range; Not detected (white bars), Not quantified (cross-hatched white bars), >0.001 <0.01 µg BMAA g^−1^ hair (light grey bars), >0.01 <0.1 µg BMAA g^−1^ hair (dark grey bars), >0.1 <1.0 µg BMAA g^−1^ hair (cross-hatched dark grey bars), >1.0 µg BMAA g^−1^ hair (black bars). The sample size (*n*) of each category is given in parentheses.

Although no overall correlation was observed between natural hair colour and the BMAA content of hairs from participants of this study, data in Figure 4a show that a greater percentage (85%) of individuals with light blonde hair had BMAA in their hair, compared to individuals with dark blonde hair (65%) and brown hair (54%). Despite these differences, no significant (α < 0.05) correlation between natural hair colour and BMAA content was observed. A much larger sample size would be required to determine whether hair colour significantly affects BMAA retention. The actual eumelanin and phaeomelanin composition of individuals’ hair can differ greatly, although the hair may appear to be the same colour [42]; therefore, the quantification of hair melanins and the correlation of hair BMAA with hair melanins may have yielded different results. The retention of BMAA by melanin-rich tissues in frogs and mice has been reported [43], and the association of BMAA with hair melanin is plausible, making hair melanin composition an additional factor that may affect accumulation of BMAA in human scalp hair. Furthermore, differences in hair care habits or the frequency of cosmetic alterations between blonde- and brown-haired individuals may also account in part for the observed difference in BMAA retention. In a simple in vitro experiment (2.1), we demonstrated that following exposure to BMAA-containing water, rough-textured dark brown hair that had been frequently cosmetically manipulated retained considerably more BMAA compared to virgin blonde hair, confirming that hair treatments, texture, age, and possibly colour may individually and in combination play an important role in the absorption of environmental BMAA into human scalp hair, contributing to a high degree of variability in the amounts of BMAA present in human hair.

Banack [29] reported the increase and subsequent decrease and disappearance of BMAA over time in the scalp hair of one individual. Although no reference was made to possible sources of BMAA, it was suggested that BMAA accumulates within an endogenous neurotoxic reservoir (accounting for the gradual increase in scalp hair BMAA content over a 23-year period), from which it is released (accounting for the decrease in scalp hair BMAA content over a subsequent 26-year period). In the current study, the percentage of individuals that tested positive for scalp hair BMAA did not differ much between individuals 19–25 years old (64%), 26–35 years old (70%), 36–65 years old (64%), or those older than 65 years (64%), as shown in Figure 4b. Statistical analysis (as described in Section 4.4) of these data showed no significant (α < 0.05) difference between age group and BMAA hair content. These data, therefore, appear to contradict the findings of Banack et al. [29], for their hair samples from a single donor over time. Many other factors that change with age, like hair care habits and lifestyle habits, are likely to affect the accumulation of BMAA in scalp hair. 

Furthermore, although Banack [32] demonstrated that orally-ingested BMAA is deposited in the hair of non-human primates in a dose-dependent manner, other studies on narcotic absorption in human hair revealed that although some evidence exists supporting a correlation between intake dose and the amount deposited into the hair shaft, there is no clearly-established correlation between intake and concentration of toxicants in hair [35]. It is therefore important to note that although the presence of BMAA in human scalp hair confirms exposure, the absence thereof does not necessarily mean the absence of exposure. 

## 3. Conclusions

BMAA could be detected in the scalp hair of 63% of all individuals tested, and the presence of BMAA appeared to correlate positively and significantly (*p =* 0.0055) to one reported hypothesised exposure route, the consumption of shellfish, and perhaps also to the frequency of consumption of pork, a previously unreported potential source of BMAA. No statistically significant correlation was observed between hair BMAA content and residential proximity to a water body with a persistent cyanobacterial bloom. Since this possible exposure site is almost constantly downwind of this reservoir, the absence of a relatively consistent BMAA content in those living within the aerosolisation zone of the shoreline suggests that other factors play a much larger role in determining the BMAA content of hair. Furthermore, the presence of BMAA in human scalp hair can be affected by an array of factors (hair care habits, hair colour, and hair structure) that are independent of BMAA exposure. This makes the analysis of the BMAA content of human scalp hair as a gauge of BMAA exposure unreliable, as the absence of BMAA does not necessary equate to the absence of exposure, nor does the amount present in the hair indicate exposure dose. To accurately use hair BMAA content as an indicator of BMAA exposure, the potential influences of these described factors on hair BMAA absorption and retention should first be defined and quantified. Given these findings, the presence of BMAA in the hair of ALS patients should also not be considered a link between BMAA and sND. Therefore, although the convenience of using hair as an indicator of BMAA exposure is appealing, care should be taken when interpreting and drawing conclusions from results, or attempting to link BMAA in hair to any pathology.

## 4. Materials and Methods

### 4.1. Study Site

The HBD reservoir is located in the North West province of South Africa. The impoundment is notorious for regular prolific algal blooms and scum events, dominated by the buoyant, toxin-producing cyanobacteria *Microcystis* [44]. The extremely dense and unfavourable algal blooms are frequent and consistent, and densest during the summer months (March–April), with remote sensing data showing 48.6% (ten-year average value, 2002–2012) cyanobacteria water surface area coverage [44]. These algal blooms are driven by the region’s warm climate, excessive nutrient loading of the water by agricultural runoffs, and poor sanitation infrastructure of informal settlements surrounding the impoundment and throughout its catchment area. 

The HBD reservoir is mainly used as a domestic and agricultural water source, with irrigation networks supplying irrigation water to approximately 162 km^2^ of agricultural land, directly or through controlled irrigation schemes and canal networks. Agriculture is mainly tobacco, vegetable crops, and wheat. Water from the dam is also used domestically in some, but not all areas of Hartbeespoort, a small resort town of 125.89 km^2^ and a population of 22,374 (density of 180 km^−2^). The town is a popular residential area, with both permanent residents and holiday homes [37,45]. 

Evaluation of HBD algal bloom toxicity has been dominated by microcystin analysis, and very few investigations have included analysis of cyanobacterial neurotoxin BMAA. Scott et al. [38], reported the presence of BMAA in 70% of phytoplankton samples taken from the dam during a bloom event in 2013, and noted an inverse relationship between BMAA and microcyctin cellular content, as well as a negative correlation between BMAA content and total available combined nitrogen. BMAA has also been reported to be present in aerosol, collected on the water or along the shoreline [21], and in fish harvested from the dam [46]. The dam is popular for water sports (water skiing, swimming, boating) and angling, with many local residents regularly consuming fish caught in the dam [46]. 

### 4.2. Hair Samples and Questionnaires 

#### 4.2.1. Ethical Statement

Human scalp hair, questionnaire data collection, and all data analysis were performed in accordance with the Declaration of Helsinki, the Belmont Report on Ethical Principles and Guidelines for the Protection of Human Subjects of Research, and, the South African National Health Act (61/2003). Ethics clearance for the collection of human scalp hair samples and questionnaires from volunteer participants was obtained from the Nelson Mandela University Human Ethics Committee prior to the commencement of this study (Ethics clearance certificate number H16-SCI-BCM-001).

#### 4.2.2. Questionnaires

Questionnaires, accompanied by a preamble explaining the relevance of the study and the conditions of voluntary participation, were designed to collect relevant data on participants’ lifestyle, dietary and smoking habits, alcohol intake, pre-existing health conditions of participant and immediate family or household members, residence and work place proximity to the water, age, gender, recreational activities (specifically those that make use of the reservoir), consumption of fish sourced from the reservoir, and consumption of locally grown fruits and vegetables (irrigated with water from the reservoir). Care was given to avoid leading questions, and all questions, except age, gender, and yes or no questions, were designed to allow numerical grading and recording of answers. Where children were involved, questionnaires were modified to be simpler, and written parental consent was obtained. All participants completed the interview-administrated questionnaires following written consent. In order to maintain the anonymity of participants, no residential addresses were collected in the questionnaires; instead, participants were provided with a map of the reservoir and its surrounding areas. The map was divided into four quadrants and contained coloured metered zones radiating away from the reservoir shoreline, each zone representing a specific distance from the reservoir shoreline. Individuals were asked to indicate in which quadrant and within which metered zone they resided and worked. Residential or workplace locations were then assessed based on the quadrant, which indicated downwind or upwind locations, and metered zones, which indicated proximity to the water. 

#### 4.2.3. Hair Sample Collection

A hair sample, consisting of the entire length of the individual’s hair, was cut with stainless steel scissors, from against the scalp and from any discrete area of the head (following written consent). The lock was secured and bound together at the non-scalp end with self-adhesive tape, on which the direction of hair growth was indicated with an arrow. Hair samples were individually stored in a sealed polyethylene bag and labeled with a number, corresponding to the associated questionnaire. All hair samples were stored at −20 °C until further processing and analysis. Hair samples were collected as described here over a 5-day period during March 2016 (a summer month).

### 4.3. Hair Sample Analysis

#### 4.3.1. Hair Sample Pre-Treatment

Exogenous hair care product residues, oils and lipids, and metal ions commonly present in hair dyes may be present on hairs, and interfere with downstream analysis. To avoid analytical interference by such compounds, a suitable and effective pre-analysis washing protocol was designed, based on previously reported solvents used in hair washing prior to analysis of environmental or narcotic contaminants. Protocols were evaluated, and the optimal solvent combination was chosen, based on its chemical potential for removing exogenous oils, lipids, hair care product residues, and metal ions, while having minimal impact on the amino acid profile of hair hydrolysates. To evaluate various hair washing protocols, virgin hair (had never received any hair care products other than shampoo or conditioner) and treated hair (frequently cosmetically altered) were donated by two female volunteers, and divided into six samples each. Each sample was then washed in a glass vial, using a different washing protocol, as detailed here, where a wash was achieved by continuous vortexing in the respective solvent for 1 min. The solvents each had a unique composition: (1) 100% methanol, distilled water [47]; (2) chloroform/acetone/methanol (1:1:1, by volume), distilled water [48]; (3) 100% methanol, chloroform/acetone/methanol (1:1:1, by volume), distilled water; (4) 5% EDTA in water, distilled water; (5) 5% EDTA in water, distilled water (×2), chloroform/acetone/methanol (1:1:1, by volume); (6) distilled water. Hair samples were thereafter dried in an oven at 45 °C, weighed and hydrolysed in 6 N HCl at 110 °C for 17 h, after which hydrolysates were filtered (Ultrafree-MC^®^ 0.22 mM centrifugal filtration units, EMD Millipore Corporation, Billerica, MA, USA), dried (Savant SpeedVac^®^, Savant Instruments Inc., Holbrook, NY, USA), and residues re-suspended in 20 mM HCl for amino acid analysis. Hair hydrolysate amino acid compositions were then determined and compared, following pre-column derivatisation with 6-aminoquinolyl-*N*-hydroxysuccinimidyl carbamate (AQC), according to the manufacturer’s instructions (Waters Ultra AccQ-Tag derivatization kit for amino acid analysis), using a Waters Acquity Ultra Performance Liquid Chromatograph (UPLC) fitted with a photodiode array (PDA). Amino acids were separated using a Waters UltraTag C_18_ column (2.1 mm × 50 mm × 1.7 µm) at 60 °C, with gradient elution (0–0.54 min, 99.9% B; 5.74 min, 90.9% B; 7.74 min, 78.8% B; 8.04 min, 40.4% B; 8.05 min, 10% B; 8.64 min, 10% B; 8.73 min, 99.9% B; 9.50 min, 99.9% B), using mobile phases specified by the Waters Ultra AccQ-Tag kit at a flow rate of 0.7 mL min^−1^. No significant differences in amino acid composition of hairs were observed between hairs washed with just water and those washed with the most stringent washing protocol (5% EDTA in water, distilled water (×2), chloroform/acetone/methanol, 1:1:1, by volume). The latter washing protocol was therefore chosen for use in all subsequently hair sample preparations, as it would ensure removal of potential interfering exogenous substances on hairs.

#### 4.3.2. BMAA Analysis

From each original hair sample, approximately 10 mg of hair was taken from the first 10 cm from the scalp end of the hair sample. On average, human scalp hair grows approximately 1 cm per month, therefore 10 cm of hair from the scalp corresponds to hair growth over the last 10–12 months. Each 10 mg sample was washed in consecutive washes with 5% EDTA in water, distilled water (×2), and chloroform/acetone/methanol (1:1:1, by volume) as described in Section 4.3.1. Washed hair was dried after which approximately 5 mg was hydrolysed, as described in Section 4.3.1. Hair samples were spiked with isotopically labelled BMAA (BMAA-4,4,4-d_3_,^15^N_2_) at 0.5 µg g^−1^ dry weight (DW) hair prior to hydrolysis.

Dried filtered hair hydrolysates were reconstituted, and diluted in 20 mM HCl. Samples were analysed for total BMAA content, as described previously [15], with one minor modification: deuterated BMAA (0.2 mg g^−1^ DW) was added to diluted samples post-hydrolysis, prior to derivatisation. Derivatised samples therefore contained two isotopically labelled internal BMAA standards (pre- and post-hydrolysis) that were used in the identification and quantification of native BMAA in hair samples. BMAA was identified and distinguished from three confounding isomers—2-aminoethylglycine (AEG), 2,4-diaminobutyric acid (DAB), and β-Amino-*N*-methyl-alanine (BAMA)—and quantified, based on retention times as well as general and diagnostic ion transitions, as previously described [15], with the addition of the internal standard BMAA-4,4,4-d_3_,^15^N_2_ transition 464.18 > 124.08 as the internal standard for quantification. The lower limits of detection and quantification of BMAA within a hair hydrolysate matrix were estimated to be 0.003 µg g^−1^ (DW) (S/N > 3) and 0.006 µg g^−1^ (DW) (S/N > 15), respectively.

In order to minimise subjective bias, all investigators were blinded during the analysis of BMAA content of hair hydrolysates. Hair samples were identified by a number and BMAA results were only correlated to corresponding questionnaire data once all BMAA analyses were completed.

#### 4.3.3. Surface Binding of BMAA

In order to establish whether the presence of BMAA in hair may be due to associations between BMAA and the external surface of the hair shaft, as well as whether such associations may be greater in cosmetically altered hair compared to virgin hair, frequently cosmetically altered (naturally brown) and virgin hair (naturally blonde) samples donated by two female volunteers were washed with water containing BMAA (50 µM). Hair samples (three replicates for each hair type) were placed individually in glass vials with BMAA-containing water for 5 min, with intermittent agitation via vortexing to simulate human hair washing. Hair was then dried in an oven at 45 °C. Hair samples then underwent the same washing regime as for other hair samples, and BMAA was identified and quantified as described in the Section 4.3.2.

### 4.4. Statistical Analysis

Using IBM^®^ SPSS^®^ Statistics software (Version 24, IBM^®^, Armonk, NY, USA), the Fischer’s exact test (two-tailed) with a significance value of α < 0.05 was used to determine whether the BMAA content of hairs was significantly different between groups of variables. Pair-wise comparisons were made between groups, and the Bonferroni correction for multiple comparisons applied where comparisons were independent [49].

## Figures and Tables

**Table 1:** Distribution of data showing total scalp hair β-*N*-methylamino-l-alanine (BMAA) content, as well as participant age, gender, and ethnicity.

Hair BMAA Content (µg g^−1^)	Gender	Age	Ethnicity	Hair BMAA Content (µg g^−1^)	Gender	Age	Ethnicity
ND	F	47	C	ND	F	25	C
ND	F	72	C	ND	F	63	C
ND	F	63	C	ND	F	58	C
ND	F	40	C	ND	F	55	C
ND	F	85	C	ND	F	62	C
ND	F	55	C	ND	F	48	C
ND	F	33	C	ND	F	20	C
ND	F	37	C	ND	F	24	C
ND	F	75	C	ND	F	33	C
ND	F	60	C	ND	F	43	C
ND	F	40	C	ND	F	47	C
ND	F	58	C	ND	F	51	C
ND	F	28	C	ND	F	72	C
ND	F	35	C	ND	F	34	C
ND	F	61	C	ND	F	44	C
ND	M	48	C	0.021	F	26	C
ND	F	25	C	0.025	M	23	C
ND	F	33	C	0.028	F	83	C
<LOQ	F	25	C	0.032	M	25	C
<LOQ	F	50	C	0.033	F	45	C
<LOQ	F	44	C	0.035	F	12	C
<LOQ	F	50	C	0.045	F	26	C
<LOQ	F	45	C	0.051	F	54	C
<LOQ	F	26	C	0.064	F	21	C
<LOQ	F	34	C	0.077	F	49	C
<LOQ	F	31	C	0.080	M	58	C
<LOQ	F	32	C	0.081	F	52	C
<LOQ	F	45	C	0.084	F	73	C
<LOQ	F	35	C	0.088	F	62	C
<LOQ	F	45	C	0.092	F	77	C
<LOQ	F	63	C	0.098	M	3	C
<LOQ	M	26	C	0.107	F	22	A
0.006	F	48	C	0.112	F	31	A
0.007	M	79	C	0.112	F	21	C
0.008	F	34	C	0.128	F	27	C
0.009	F	27	C	0.149	F	63	C
0.012	M	50	C	0.226	F	54	C
0.014	F	50	C	0.240	F	69	C
0.015	M	18	C	0.695	F	36	C
0.016	F	26	C	0.723	F	24	C
0.016	F	43	C	0.741	F	43	C
0.016	F	52	C	1.164	M	67	C
0.018	F	61	C	1.652	F	35	C
0.021	F	47	C	9.636	F	54	C

ND = not detected, <LOQ = trace amount of BMAA detected but below limit of quantification, M = Male, F = Female, C = Caucasian, A = African.
